# Case Report: Central nervous system infiltration after allogeneic hematopoietic stem cell transplantation in high-risk myelodysplastic neoplasms

**DOI:** 10.3389/fonc.2026.1916682

**Published:** 2026-09-07

**Authors:** Lejian Shi, Jinjin Zhu, Yuhang Wang, Depei Wu, Miao Miao, Huifen Zhou, Huanle Gong

**Affiliations:** 1 National Clinical Research Center for Hematologic Diseases, Jiangsu Institute of Hematology, The First Affiliated Hospital of Soochow University, Suzhou, China; 2 Institute of Blood and Marrow Transplantation, Collaborative Innovation Center of Hematology, Soochow University, Suzhou, China

**Keywords:** allogeneic hematopoietic stem cell transplantation, central nervous system leukemia, chronic myelomonocytic leukemia, CNS infiltration, myelodysplastic neoplasms, TP53

## Abstract

**Background:**

Although central nervous system leukemia (CNSL) is a recognized complication of acute leukemia, myelodysplastic neoplasms (MDS) complicated by CNSL are exceedingly rare, particularly after allogeneic hematopoietic stem cell transplantation (allo-HSCT). Previous studies have largely focused on chronic myelomonocytic leukemia with central nervous system (CNS) involvement. Here, we describe the first comprehensively documented case of isolated CNS infiltration after allo-HSCT in a patient with MDS.

**Case presentation:**

A patient with high-risk MDS with increased blasts-2, harboring *IDH1* and *TP53* mutations, a complex karyotype, and a very high Molecular International Prognostic Scoring System score underwent unrelated allo-HSCT. On day +19 post-transplantation, the patient developed a severe, unexplained headache. Cerebrospinal fluid (CSF) analysis by flow cytometry revealed blasts largely immunophenotypically consistent with those in the bone marrow at initial diagnosis, while concurrent bone marrow assessment confirmed remission. CSF blasts were cleared following triple intrathecal therapy (IT). To date, the patient remains in remission on azacitidine maintenance therapy, with an overall survival of 14 months from diagnosis.

**Conclusion:**

Although CNSL is exceptionally rare in patients with MDS, this case demonstrates that isolated CNS infiltration may occur after allo-HSCT. Our findings emphasize that CNS involvement should be considered in the differential diagnosis of patients with MDS presenting with neurological manifestations, even without marrow relapse. Furthermore, this case highlights the critical role of CSF flow cytometry in establishing the diagnosis when conventional CSF studies are inconclusive.

## 1 Introduction

MDS are a heterogeneous group of clonal myeloid disorders driven by acquired genomic mutations in hematopoietic stem cells. They are characterized by persistent cytopenias, dysplastic hematopoiesis, and a substantial risk of progression to acute myeloid leukemia (AML) (1). For patients with high-risk MDS, allo-HSCT remains the only potentially curative intervention and should be pursued whenever feasible. In patients who are not immediately eligible for transplantation, bridging strategy with hypomethylating agents combined with venetoclax may be considered to reduce disease burden before allo-HSCT (2, 3).

While CNSL is a well-recognized and common therapeutic challenge in acute leukemia, its association with classic MDS is uncommon. Previous studies of CNS involvement have predominantly focused on chronic myelomonocytic leukemia (CMML) with myeloproliferative features (4–14). Beyond the single reported case of CNSL at MDS diagnosis (15), we report a comprehensively documented case of isolated CNS infiltration after allo-HSCT in MDS with increased blasts-2 (MDS-IB2), confirmed by CSF flow cytometry on day +19.

## 2 Case description

A 38-year-old woman presented with a one-week history of oral blood blisters and subcutaneous bleeding. Her medical history was significant only for chronic migraines. Laboratory evaluation revealed pancytopenia with a white blood cell count (WBC) of 1.34 × 10^9^/L, absolute neutrophil count of 0.25 × 10^9^/L, hemoglobin of 85 g/L, and platelet count of 21 × 10^9^/L. During the initial hospitalization, she experienced allergic reactions to both platelet transfusion and the antibiotic piperacillin-tazobactam.

**Figure 1:**
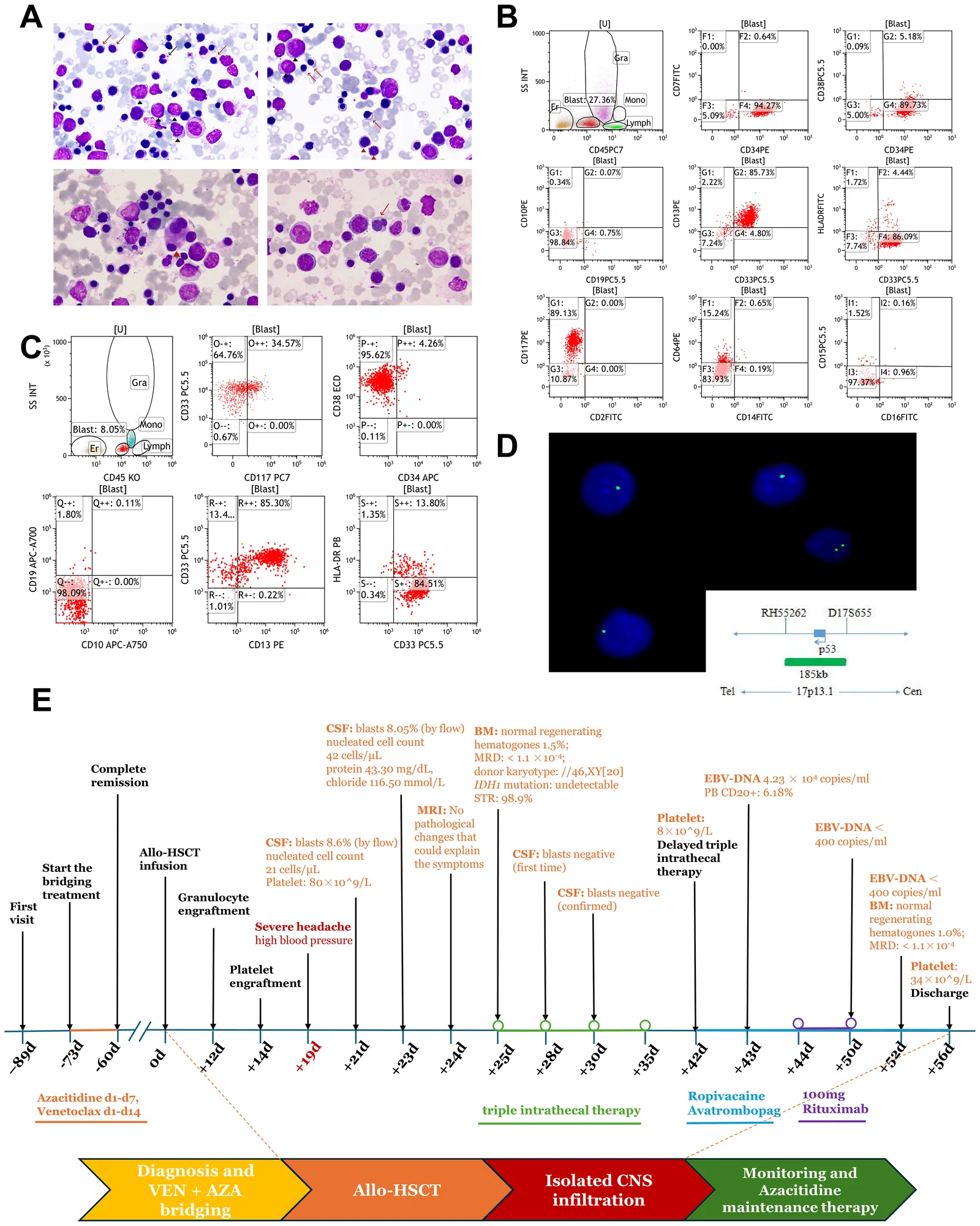
**(A)** Initial bone marrow cytology. Wright-Giemsa-stained bone marrow smear was examined at ×100 magnification. In the upper left and upper right panels, the red arrow indicates a cytoplasmic vacuole in an erythroid cell, the black triangle marks a blast, and the black arrow points to a pseudo-Pelger–Huët anomaly. The red triangle indicates a megaloblastoid erythroblast. In the lower left panel, the red triangle indicates a multinucleated megakaryocyte. In the lower right panel, the red arrow indicates a binucleated erythroid precursor. **(B)** Initial bone marrow flow cytometry. After doublet exclusion, the CD45 vs. side scatter (SSC) plot shows the following gated populations: Er (Erythrocyte), Lymph (lymphocytes), Mono (monocytes), Gra (granulocytes), and blasts (red, 27.36%). The blast population showed the following antigen expression: CD34^+^CD117^+^CD13^+^CD33^+^. **(C)** Cerebrospinal fluid flow cytometry. After doublet exclusion, the CD45 vs. SSC plot shows the following gated populations include Er (Erythrocyte), Lymph (lymphocytes), Mono (monocytes), Gra (granulocytes), and blasts (red, 8.05%). Blasts showed the following antigen expression: CD34^-^CD117 ± CD13^+^CD33^+^CD38^+^HLA-DR^+^CD45±. Compared with **(B)**, CD34 expression is decreased, while CD38 expression is increased. **(D)** FISH for *TP53*. Single-color FISH for *TP53* on the diagnostic bone marrow specimen (methanol–acetic acid fixed, −20 °C), performed retrospectively after CNS infiltration was identified. Original magnification ×100. Most nuclei showed a single green signal; enumeration of 300 interphase nuclei revealed a *TP53* deletion in 96% of nuclei. **(E)** Timeline of symptoms, laboratory tests, and treatment. Orange, black, and red text denote key test results, important events, and the onset of CNS infiltration, respectively. Treatment measures are listed below the timeline, while the four blocks at the bottom outline the diagnostic and therapeutic stages.

Bone marrow examination confirmed MDS-IB2 with 10% myeloblasts and dysplasia in all three lineages (erythroid, granulocytic, and megakaryocytic), each exceeding 10%, with no Auer rods present (**Figure 1A**). Granulopoiesis accounted for 11.50% of all nucleated cells, with impaired nuclear lobulation, hypogranulation, and nuclear-cytoplasmic dyssynchrony. Erythropoiesis was expanded (69.50%), exhibiting nuclear budding, binucleation, cytoplasmic vacuolization, and megaloblastoid changes. Ring sideroblasts constituted 28% of erythroid precursors. Megakaryocytes were increased (>100/smear), with morphologies ranging from single double, or multiple round nuclei. Micromegakaryocytes were easily identified. Platelet clusters were rare. Flow cytometry revealed 27.36% myeloid blasts (CD34+, CD117+, CD13+, and CD33+) (**Figure 1B**). Both morphological and flow cytometric calculations used total nucleated cells as the denominator, which includes nucleated red blood cells. This discrepancy most likely stems from a substantial difference in nucleated red blood cells content in the denominator (69.5% vs. 16.6%). Cytogenetic analysis revealed a complex karyotype, including der(5;17)(p10;q10), with complete karyotypic details provided in **Table 1**. Next-generation sequencing (NGS) identified *IDH1* p.Arg132Cys mutation with a variant allele frequency (VAF) of 4.0%. No pathogenic mutations, including *TP53* mutations, were detected by RNA sequencing. The patient was a heterozygous carrier of a pathogenic *FANCM* truncating variant (NM_001018113.3:c.543G>A, p.Trp181*; VAF 50.87%) and a *UGT1A1* variant (NM_000463.3:c.211G>A, p.Gly71Arg; VAF 53.69%). Her IPSS-M score was 1.91 (very high risk). At diagnosis, serum lactate dehydrogenase (LDH) was within the normal range, and monocytosis was absent (peak absolute monocyte count 0.31 × 10^9^/L). Clinical and laboratory parameters at pivotal time points are listed in **Table 1**.

**Table 1:** Key clinical and laboratory parameters at critical time points.

Parameters	At diagnosis (May 2025)	Before transplantation, after bridging (August 2025)	Central nervous system infiltration (September 2025)	Last follow-up (July 2026)
Clinical presentation	Oral blood blisters and subcutaneous bleeding	Complete remission	Refractory headache, hypertension	Complete remission
WBC (×10^9^/L)	1.34	5.11	5.79	6.6
ANC (×10^9^/L)	0.25	3.81	3.89	2.06
HGB (g/L)	85	118	105	119
PLT (×10^9^/L)	21	154	78	198
Bone Marrow cytology	10% myeloblasts	1% normal regenerating hematogones	1.50% normal regenerating hematogones	2.50% normal regenerating hematogones
Bone Marrow minimal residual disease	CD34+, CD117+, CD13+, CD33+HLA-DR+ 27.36%	CD34+CD117+CD13+CD33+CD7+CD10-CD19-CD38+HLA-DR+CD45± <1.5X10^-4^	<1.1X10^-4^	<9.2X10^-4^
*IDH1* p.Arg132Cys VAF	Present 4.00%	Present 0.80%	Undetectable	Undetectable
*TP53* alterations	No *TP53* sequence mutation detected by RNA-seq; but *TP53* deletion in 96% of interphase nuclei by FISH	Not assessed	Not assessed	Negative for *TP53* deletion by FISH
Cytogenetics	44,xx,?der(5;17)(p10;q10),?der(7;12)(p10;q10)[10]	Not assessed	A donor-derived male karyotype, 46,XY[20]	Predominantly donor-derived (46,XY [20]), but one metaphase carried a derivative chromosome: 46,XY,?dic (11;14), +mar
Blasts in the CSF	Not assessed	Not assessed	8.6%→8.05%(by flow) (Immunophenotype: CD34–CD117+CD13+CD33+CD38+HLA-DR±)	No abnormal cells were observed
Key Interventions	Bridging therapy with azacitidine and venetoclax	Unrelated allo-HSCT	Triple intrathecal chemotherapy	Low-dose azacitidine maintenance therapy

allo-HSCT, allogeneic hematopoietic stem cell transplantation; ANC, absolute neutrophil count; FISH, fluorescence *in situ* hybridization; HGB, hemoglobin; PLT, platelet count; VAF, variant allele frequency; WBC, white blood cell count. Notes: Bold font highlights the changes in the immunophenotype of cerebrospinal fluid compared to that of the bone marrow.

Allo-HSCT was planned. As a suitable human leukocyte antigen (HLA)-matched related donor was unavailable, the patient received bridging therapy with azacitidine and venetoclax and subsequently achieved complete remission on day -60. Due to high HLA antibody levels, the patient received three sessions of immunoadsorption. Two months after diagnosis, the patient underwent allo-HSCT from an unrelated donor. The conditioning regimen comprised a modified busulfan/cyclophosphamide (BuCy) protocol with high-dose cytarabine (2 g/m²). Graft-versus-host disease (GVHD) prophylaxis included cyclosporine, mycophenolate mofetil, anti-thymocyte globulin, and short-course methotrexate. The graft comprised peripheral blood stem cells from a 9/10 HLA-matched, male, O-positive, unrelated donor to the female recipient. The infused cell doses were as follows: nucleated cells, 15.21 × 10^8^/kg, CD34^+^ cells, 8.06 × 10^6^/kg; and CD3^+^ cells, 3.29 × 10^8^/kg. Neutrophil engraftment occurred on day +12 and platelet engraftment on day +14.

On day +19 post-transplantation, the patient developed severe recurrent headaches accompanied by transient perioral numbness, prompting a neurological evaluation. Physical examination revealed bilateral conjunctival edema and elevated blood pressure, peaking at 162/110 mmHg. No focal neurological deficits or meningeal signs were identified. Cranial magnetic resonance imaging (MRI) revealed minor nonspecific white matter hyperintensities in the bilateral frontal subcortical regions (Fazekas grade 1) and sinusitis. There were no MRI features suggestive of a space-occupying lesion, intracranial hemorrhage, or the characteristic vasogenic edema associated with posterior reversible encephalopathy syndrome (PRES).

Due to persistent headache, a diagnostic lumbar puncture was performed on day +21, when the platelet count was 80 × 10^9^/L. Routine CSF analysis showed a mildly elevated nucleated cell count of 21 cells/μL (reference, <5 cells/μL). CSF cytology, biochemistry, and pathogen-targeted NGS were unremarkable. However, CSF flow cytometric analysis revealed a distinct population comprising 8.6% of total cells, with an immunophenotype of CD34^-^CD117^+^CD13^+^CD33^+^CD7^-^CD19^-^CD56^-^CD10^-^CD38^+^HLA-DR^+^CD45±. A repeat lumbar puncture on day +23 yielded a similar population (8.05%) with an identical myeloid phenotype, while concurrent cytology remained negative for blasts (nucleated cell count of 42 cells/μL, **Figure 1C**). Peripheral blood smears obtained contemporaneously with the lumbar punctures showed no blasts, making specimen contamination unlikely. The immunophenotype of the CSF blasts was largely congruent with that of the diagnostic bone marrow sample, albeit with loss of CD34 and acquisition of CD38. CSF flow cytometry was reproduced across two separate samples analyzed at different institutions. On day +24, the patient reported light menstrual flow with lower abdominal discomfort. Gynecologic ultrasound showed no abnormalities, with no evidence of concurrent involvement of the reproductive system. A concurrent bone marrow aspirate on day +25 confirmed morphological remission (1.5% normal myeloid blasts) with full donor chimerism and no detectable *IDH1* mutation. The patient was afebrile with no signs of bleeding or worsening anemia. Plasma Epstein–Barr virus (EBV) DNA was <400 copies/mL. In summary, definitive detection of myeloid blasts in the CSF by flow cytometry, in contrast to morphologic and molecular remission in the bone marrow, established the diagnosis of isolated CNS infiltration of MDS following allo-HSCT. Conventional sequencing did not detect *TP53* mutations at the time of diagnosis. However, following CNS infiltration, fluorescence *in situ* hybridization (FISH) performed on the bone marrow sample from initial diagnosis revealed a *TP53* deletion in 96% of interphase nuclei (**Figure 1D**).

On the same day (day +25), lumbar puncture was performed, followed by triple intrathecal chemotherapy with cytarabine (40 mg), methotrexate (15 mg), and dexamethasone (5 mg). Intrathecal chemotherapy was administered every other day. Blasts in the CSF became undetectable on two consecutive tests after three doses. The regimen was de-escalated to weekly administration. After four doses, treatment was suspended because of severe therapy-related thrombocytopenia (platelet count, 8 × 10^9^/L), necessitating transfusion support and growth factor therapy. Other manageable complications included EBV infection, which resolved after two doses of rituximab. During follow-up, the patient did not develop acute or chronic GVHD. See **Figure 1E** for a detailed timeline.

After complete remission, the patient received three cycles of low-dose subcutaneous azacitidine (35 mg/m²/day for 3 days) as maintenance therapy. Following the initial platelet recovery, the patient received intrathecal chemotherapy. Consolidation therapy was subsequently administered less frequently (approximately one injection every 4 months) due to thrombocytopenia associated with concurrent azacitidine maintenance and other contributing factors. At the last follow-up, the patient remained alive, with an overall survival of 14 months from diagnosis and 10 months from CNS infiltration. The most recent bone marrow evaluation showed 2.5% normal regenerating hematogones, no detectable *IDH1* mutations and no *TP53* deletion by FISH. Notably, follow-up cytogenetic analysis revealed a new single-cell anomaly, with a predominantly donor-derived karyotype (46,XY [20]) and one metaphase harboring a derivative chromosome, 46,XY,?dic(11;14),+mar warranting close surveillance. Given the *TP53* deletion, complex karyotype, and early CNS infiltration, remission was considered fragile.

## 3 Discussion

To our knowledge, the only well-documented case of MDS with CNS involvement was a 25-year-old man who initially presented with left upper limb weakness (15). MRI revealed acute cerebral infarction and diffuse edema, with blasts detected in the CSF. A bone marrow examination confirmed the diagnosis of MDS. In contrast, our patient developed isolated CNS infiltration after transplantation, representing a different clinical scenario. Here, we present a comprehensively documented case of isolated CNS infiltration after allo-HSCT in a patient with MDS-IB2. To contextualize this rare association between MDS and CNSL, we reviewed the available literature.

This patient fulfilled the WHO criteria for MDS-IB2 (peak absolute monocyte count, 0.31 × 10^9^/L, excluding CMML) (16). Her post-transplant course was notable for severe headaches and hypertension. The differential diagnoses included PRES (17, 18), infectious meningitis or encephalitis (19), post-transplant lymphoproliferative disorder (PTLD) (20, 21), and migraine recurrence (22). These were systematically excluded based on the investigations detailed in **Supplementary Table 2**. The diagnosis ultimately rested on CSF analysis, flow cytometry identifying a myeloid blast population largely immunophenotypically consistent with the original marrow clone. This finding was confirmed in two temporally distinct CSF specimens, each independently analyzed at separate institutions. Concurrent peripheral blood smears showed no blasts, thereby excluding contamination. Critically, the bone marrow remained in morphological and molecular remission, and cranial MRI revealed no space-occupying lesions or PRES-related edema. Furthermore, her subsequent response to IT and the absence of progression to AML at the last follow-up corroborated the diagnosis of isolated CNS infiltration after allo-HSCT.

Immunophenotypic comparison revealed that the CSF blasts largely matched those in the original bone marrow aspirate. The core myeloid differentiation antigens, CD13, CD33, and CD117, were retained. However, two notable immunophenotypic shifts were observed, suggesting clonal evolution. First, the recurrent clone lost CD34 expression and showed diminished HLA-DR expression, reflecting a shift from an early stem/progenitor cell profile toward a more primitive myeloid precursor phenotype, a feature often associated with disease recurrence and drug resistance. Second, the blasts exhibited persistently strong CD38 positivity, a pattern that was already evident in the pre-transplantation bone marrow sample. CD38 was highly expressed in both early and activated hematopoietic cells (23). This sustained high expression suggests that the recurrent clone was composed of proliferative, metabolically active cells with increased invasive potential.

In the absence of a pre-transplant lumbar puncture baseline, we were unable to definitively diagnose post-transplant CNS relapse and therefore classified this as CNSL in the setting of MDS. We hypothesized that residual leukemic stem cells (LSCs) were present in the CNS prior to transplantation. First, the clinical symptoms of CNS infiltration, which emerged as early as day +19 post-transplantation, are unlikely to stem from sufficient clonal expansion from a minimal residual state to a clinically detectable level. Furthermore, studies have shown that CSF cells exhibit a donor phenotype from days 19 to 97 post-transplant (24), indicating that effective graft-versus-leukemia activity has not yet been established in the CNS. The quiescent state of LSCs may have enabled them to evade elimination by cytarabine, a drug that preferentially targets actively cycling cells (25). During the post-transplant immunosuppressive period and the window before donor cells exert graft-versus-leukemia effects, these LSCs likely contribute to isolated CNS infiltration.

In AML, established risk factors for CNSL include younger age (<64 years), elevated WBC and LDH levels at diagnosis, specific genetic lesions (e.g., *FLT3*-ITD and core-binding factor aberrations), and monocytic differentiation (25). While *IDH1* mutations can disrupt the blood-brain barrier via the oncometabolite 2-hydroxyglutarate (26), and *TP53* mutations portend poor prognosis (27, 28), neither is independently linked to CNSL. Therefore, any link observed in our patient should be interpreted with caution. Mechanistically, *TP53* dysfunction confers resistance to systemic therapy and may enhance immune evasion, potentially enabling leukemic cells to survive and proliferate within the immunologically privileged CNS. Theoretically, the co-occurrence of *IDH1* and *TP53* mutations could synergistically increase the risk of CNSL. This hypothesis warrants further investigation to elucidate driver mutation-specific mechanisms of CNS tropism and explore potential targeted strategies.

Regarding treatment, although the patient harbored an *IDH1* mutation prior to transplantation, ivosidenib has not yet been approved for MDS in China, and its use remains in the clinical trial stage (3). To achieve rapid tumor burden reduction and secure the window for transplantation (29), the patient received venetoclax + azacitidine as bridging therapy prior to transplantation, rather than azacitidine + ivosidenib. No parenchymal lesions were found on MRI, so radiotherapy was not indicated. The addition of high-dose cytarabine would have further compounded thrombocytopenia, potentially limiting the feasibility of IT. Additionally, there were deviations from guideline recommendations regarding intrathecal chemotherapy frequency. The every-other-day triple-agent intrathecal regimen exceeded the National Comprehensive Cancer Network (NCCN) recommended frequency of twice weekly, representing a more aggressive frequency within the guideline framework. However, during the subsequent consolidation period, the actual frequency was lower than the recommended weekly administration for 4–6 weeks, as the patient’s platelet count dictated the timing of consolidation treatment. This reduction in frequency may have compromised the optimal consolidation of CNSL. Close clinical follow-up and regular CSF monitoring were performed throughout the treatment course. From a prognostic perspective, patients with MDS and *TP53* alterations undergoing allo-HSCT still have a higher 2-year cumulative incidence of relapse and lower 2-year disease-free survival and overall survival, especially those with a complex karyotype. Post-transplantation MRD positivity is an independent adverse prognostic factor (27). Isolated CNS infiltration may portend a relatively more favorable prognosis than bone marrow infiltration or combined relapse.

Given the paucity of literature on CNS involvement in MDS, we expanded our review to include related cases of CMML, a myelodysplastic/myeloproliferative neoplasm that shares clinical and genetic features with MDS. We identified 13 patients with CMML and MDS with CNS involvement *(***Supplementary Materials, Table 3***)*. No consistent patterns have emerged from the available molecular genetic or cytogenetic data. Motor dysfunction was the most common symptom observed (n = 9). CNSL can be diagnostically challenging, and its imaging findings are highly variable, ranging from hematoma, meningeal enhancement, and cerebral infarction to entirely unremarkable findings. A definitive diagnosis relied on invasive procedures (e.g., lumbar puncture performed in nine cases, stereotactic biopsy (31), surgical pathology (11), or autopsy (4, 6, 13)). Once diagnosed, all patients treated with intrathecal chemotherapy responded (seven cases), whereas the prognosis was uniformly poor for the three patients who received only glycerol and steroids (4, 6, 13), highlighting the critical need for prompt and appropriate intervention. Therefore, a high index of clinical suspicion is essential for addressing this diagnostic dilemma.

Severe thrombocytopenia or poor performance status frequently precludes safe lumbar puncture, presenting a major diagnostic hurdle. This case highlights the increased reliance on advanced imaging techniques in such scenarios. In patients with CSF-positive CNSL, imaging is crucial for identifying bulky disease that may warrant additional local therapy such as radiotherapy. Conversely, in CSF-negative cases with a high clinical suspicion, MRI of the neuroaxis can sometimes detect leukemic infiltrates missed by CSF analysis (25). Furthermore, magnetic resonance spectroscopy offers additional metabolic insights. A retrospective analysis correlating MRI with pathology in CNSL concluded that, in the context of a stable creatine peak, an elevated choline or a reduced/absent N-acetylaspartate peak provides supportive diagnostic evidence, particularly when conventional MRI sequences are non-diagnostic (9). This metabolic signature may help distinguish from non-neoplastic processes. For invasive procedures, platelet counts should generally be maintained above 50 × 10^9^/L for a diagnostic lumbar puncture (32), and approximately 70 × 10^9^/L for a surgical biopsy (11). Finally, in cases of high suspicion when a definitive diagnosis remains elusive, a diagnostic/therapeutic trial should be considered. Informed by an autopsy-confirmed case of CMML with CNSL initially presenting with hemorrhagic nodules on MRI (13), one center reported that high-dose cytarabine led to sustained neurological improvement over 24 months in patients clinically diagnosed with CMML-CNSL based on MRI and exclusion of alternatives (14).

We describe the first comprehensively documented case of isolated CNS infiltration in patient with MDS following allo-HSCT, while the bone marrow remained in remission. This finding supports the possibility of CNS involvement in a patient with MDS. Retrospective analyses of AML cohorts have not demonstrated a significant reduction in CNS relapses with intrathecal prophylaxis (33, 34), suggesting that routine prophylactic strategies may have limited benefits. Accordingly, clinicians should maintain a high index of suspicion for CNS involvement in patients with MDS/CMML who present with unexplained neurological symptoms, including headache, vomiting, sensory or motor deficits, or cognitive-behavioral changes (35). When feasible and not contraindicated, a timely diagnostic workup with lumbar puncture (including CSF flow cytometry for immunophenotyping) and neuroimaging is essential. The differential diagnosis should also encompass other early neurological complications after allo-HSCT, such as infection, cerebrovascular events, GVHD, treatment-related neurotoxicity, and PTLD (36). In addition, the co-occurrence of *IDH1* and *TP53* mutations in this patient may have predisposed her to aggressive CNS tropism, warranting further investigation into the mutation-specific mechanisms of CNS infiltration.

This study had several limitations. Given the rarity of CNS involvement in MDS (reported only in case reports) and the absence of routine screening recommendations for asymptomatic patients (NCCN AML v5.2026 (30); no analogous MDS guidelines exist), we did not perform pre-transplantation lumbar puncture. This limitation prevented us from distinguishing whether CNS involvement represented pre-existing infiltration or post-transplant recurrence. Another important limitation is the use of natural sedimentation rather than a cytospin preparation for CSF cytology. Natural sedimentation has a lower reported sensitivity for detecting malignant cells than cytospin preparation (19.0% vs. 27.8%) (37), which may explain the discrepancy between morphological and flow cytometric findings. Future studies should focus on cytospin preparations to address these limitations.

## 4 Conclusion

In summary, we describe the first comprehensively documented case of isolated CNS infiltration following allo-HSCT for MDS. These findings highlight the importance of considering CNS infiltration in the differential diagnosis for any MDS patient with unexplained neurological symptoms. Future cases of this nature could benefit from the molecular and cytogenetic analyses of CSF samples, where clinically appropriate or under a research protocol, to deepen insights into CNS tropism in myeloid malignancies.

## 5 Patient perspective

In addition to the standardized medical records, the patient recounted her experience of diagnosis and treatment as follows: “When I first heard the news, it struck me like a bolt from the blue. I was devastated, sinking into a place so dark that I considered giving up. Just as I had gotten through the transplant and began to feel a flicker of hope, sudden CNS infiltration plunged me back into despair. For two days, I was consumed by panic and dread, too afraid to tell my family the truth about my condition. However, the reality must be faced. Fortunately, after my first IT, the doctor’s words rekindled my hope: the blasts were no longer detected. Since then, I have fully cooperated with my doctors and undergone lumbar punctures every three months. The blasts remained undetectable. This journey taught me one invaluable lesson: never give up on yourself, no matter what.

## Data Availability

The raw data supporting the conclusions of this article will be made available by the authors, without undue reservation.

## Supplementary material

Supplementary Table 2 Differential diagnosis and key evidence for exclusion.

Supplementary Table 3 Medical records of 13 patients with MDS/CMML with CNSL can be viewed in Excel.
